# ^68^Ga-PSMA PET/CT in radioactive iodine-refractory differentiated thyroid cancer and first treatment results with ^177^Lu-PSMA-617

**DOI:** 10.1186/s13550-020-0610-x

**Published:** 2020-03-06

**Authors:** Lisa H. de Vries, Lutske Lodewijk, Arthur J. A. T. Braat, Gerard C. Krijger, Gerlof D. Valk, Marnix G. E. H. Lam, Inne H. M. Borel Rinkes, Menno R. Vriens, Bart de Keizer

**Affiliations:** 1 Department of Surgery, University Medical Centre Utrecht, Heidelberglaan 100, 3584 CX Utrecht, The Netherlands; 2 Department of Radiology and Nuclear Medicine, University Medical Centre Utrecht, Heidelberglaan 100, 3584 CX Utrecht, The Netherlands; 3 Department of Endocrine Oncology, University Medical Centre Utrecht, Heidelberglaan 100, 3584 CX Utrecht, The Netherlands

**Keywords:** Radioactive iodine-refractory differentiated thyroid carcinoma, Prostate-specific membrane antigen, Theranostic, Gallium, Lutetium, PET/CT

## Abstract

**Background:**

Differentiated thyroid carcinoma (DTC) is the most common type of thyroid cancer. Treatment with surgery, radioactive iodine (RAI), and TSH suppression is effective in most patients. Five to 15% of patients become RAI refractory and need alternative therapy; however, treatment options are limited. ^68^Ga-PSMA PET/CT, originally developed for prostate cancer, is also applicable to other malignancies, including thyroid carcinoma. The uptake of PSMA in thyroid carcinoma gives opportunities for imaging and therapy of RAI-refractory DTC. The aim of this study was to analyze imaging on ^68^Ga-PSMA PET/CT and evaluate the response to ^177^Lu-PSMA-617 therapy in patients with RAI-refractory DTC.

**Materials and methods:**

Five patients with RAI-refractory DTC underwent ^68^Ga-PSMA PET/CT to determine their eligibility for ^177^Lu-PSMA-617 therapy. ^68^Ga-PSMA PET/CTs were analyzed visually and quantitatively. Response to ^177^Lu-PSMA-617 therapy was evaluated using imaging and thyroglobulin (Tg) values.

**Results:**

Tracer uptake suspicious for distant metastases was depicted in all ^68^Ga-PSMA PET/CTs. Based on tracer uptake, three patients were eligible for ^177^Lu-PSMA-617 therapy, of whom two were treated. One patient showed disease progression on imaging 1 month later, while her Tg values gradually increased from 18 to 63 μg/L in the months after treatment. Another patient showed partial, temporary response of lung and liver metastases. Her Tg levels initially decreased from 17 to 9 μg/L. However, 7 months after treatment, there was disease progression on imaging and Tg levels had increased to 14 μg/L.

Imaging with ^68^Ga-PSMA PET/CT could be compared to ^18^FDG PET/CT in three patients. Two patients showed additional lesions on ^68^Ga-PSMA PET/CT, and one patient showed concordant imaging.

**Conclusion:**

^68^Ga-PSMA PET/CT appears to have added value in patients with RAI-refractory DTC, as it is able to detect various types of lesions, some of which were not picked up by ^18^FDG PET/CT. Furthermore, ^68^Ga-PSMA PET/CT might be used to identify patients eligible for treatment with ^177^Lu-PSMA-617. One of the two patients who underwent ^177^Lu-PSMA-617 therapy showed a modest, temporary response. To draw conclusions about the effectiveness of this therapy, more research is needed.

## Introduction

Differentiated thyroid carcinoma (DTC) is the most common (> 90%) type of thyroid carcinoma [1]. Treatment with surgery, radioactive iodine (RAI), and TSH suppression is effective in most patients, resulting in 10-year survival rates of over 90%. Less than 10% of patients develop distant metastases, and 5–15% of patients become RAI refractory [2, 3]. These patients do not take up RAI in (a part of) the lesions on diagnostic ^131^I scan or after ^131^I therapy or show progression of metastases despite substantial RAI uptake or cumulative ^131^I activity of > 22.2 GBq [4]. For these patients, the prognosis is poor as 10-year survival rates decrease to 10%, from the time metastases are detected [2].

Patients with RAI-refractory disease need alternative therapy, but treatment options are limited. Currently, for patients with asymptomatic, stable, or minimally progressive disease, monitoring while on TSH suppression is advised by the 2015 American Thyroid Association management guidelines [1]. Interventions should only be used to prevent morbidity or palliate symptoms. In case of threatening or symptomatic lesions, directed therapy such as surgery, radiation, or peptide receptor radionuclide therapy (PRRT) is an option. Systemic therapy such as with tyrosine kinase inhibitors is another alternative (TKIs). However, due to severe side-effects, TKIs can decrease quality of life and should be considered carefully [1]. Hence, there is a demand for new treatment modalities for patients who suffer from RAI-refractory DTC.

Prostate-specific membrane antigen (PSMA) is a type II transmembrane glycoprotein receptor, expressed in normal prostate tissue and to a greater extent in prostate carcinoma, making it a useful target for radionuclide imaging and therapy for prostate cancer [5–7]. PSMA is also expressed in the neovasculature of other tumors including carcinomas of the lung, breast, colorectum, and thyroid [8–12]. Compared to normal thyroid tissue, PSMA is significantly overexpressed in the neovasculature of DTC, especially in the RAI-refractory type [13]. Altogether, PSMA is a potential target for theranostics in RAI-refractory DTC. Lütje et al*.* and Verma et al*.* found results suggesting that the use of ^68^Ga-PSMA PET/CT in DTC is suitable for determining whether a RAI-refractory patient is eligible for PSMA-targeted therapies, such as treatment with ^177^Lu-PSMA-617 [14, 15].

To investigate the value of the theranostic PSMA, we performed a single-center retrospective analysis on patients with RAI-refractory DTC, referred for ^68^Ga-PSMA PET/CT to investigate suitability for peptide radioligand therapy with ^177^Lu-PSMA-617.

## Methods

### Patient selection

The multidisciplinary tumor board, consisting of at least one surgeon, endocrinologist, pathologist, radiologist, and nuclear medicine physician, decided whether patients with RAI-refractory DTC should undergo a ^68^Ga-PSMA PET/CT to determine their eligibility for therapy with ^177^Lu-PSMA-617. The ^68^Ga-PSMA PET/CTs made between 2016 and 2019 were retrospectively analyzed. Written informed consent was obtained from all living patients.

### Patient characteristics

Patient characteristics including gender, age, treatment history, presence of locoregional recurrence/metastases or distant metastases, Tg antibodies, Tg levels, and TSH levels were retrieved from the medical files. An immunoradiometric assay (IRMA) (Brahms, Hennigsdorf, Germany) was used for measuring Tg antibodies and Tg until July 2017 and October 2017, respectively. Ever since, a chemiluminescence assay was used on a Liaison analyzer (DiaSorin, Saluggia, Italy). The functional sensitivity was 0.2 μg/L for both Tg assays. Patient characteristics are provided as median and range.

### Preparation of radiopharmaceuticals

^68^Ga-PSMA-11 and ^177^Lu-PSMA-617 were prepared according to Good Manufacturing Practice using a fully automated system for routine production of radiopharmaceuticals and accompanying reagent kits and cassettes per manufacturer’s recommendations (Modular Lab Easy, Eckert & Ziegler, Berlin, Germany). Eppendorf tubes with 40 μg PSMA-11 or PSMA-617 were defrosted from − 20 °C storage and used for batch preparations (ABX, Radeberg, Germany). A ^68^Ge-^68^Ga-generator (GalliaPharm, Eckert & Ziegler, Berlin, Germany) and ^177^LuCl3 (EndolucinBeta, ITG, Garching, Germany) were used for radiolabeling.

Small aliquots (20–50 μL) were withdrawn from end products for quality controls. Thin-layer chromatography (TLC), high-performance liquid chromatography (HPLC), and pH, endotoxin, and sterility determinations were used for radiochemical purity as described previously [16, 17].

### Image acquisition and analysis

^68^Ga-PSMA-11 was administered intravenously in a dose of 1.5–2 MBq/kg. Combined PET and CT images were performed approximately 60 min after injection, from the skull vertex to the thighs using a TruePoint Biograph mCT40 scanner (Siemens, Erlangen, Germany). A low-dose CT scan was performed using Care Dose 4D and Care kV, with the following reference parameters: 40 mAs and 120 kV. PET was acquired according to the European Association of Nuclear Medicine recommendations with the following parameters: PET with time-of-flight and point spread function (TrueX) reconstruction, four iterations, 21 subsets, with a filter of 7.5 mm full width at half maximum [18]. For standardized uptake values (SUV) measurements, the lean body mass corrected values were used.

^68^Ga-PSMA PET/CT scans were collected and reviewed by a dedicated board-certified head and neck nuclear medicine physician (BdK) experienced in ^68^Ga-PSMA PET/CT. On each scan, pathological focal tracer uptake was assessed and SUV measurements were performed on representative metastases. This included both the region of the (former) primary tumor, as well as regional or distant metastasis. Maximum SUV (SUV_max_) were measured using a freehand isocontour volume of interest in these areas, using Syngo.via (Siemens, Erlangen, Germany). Also, all ^18^FDG PET/CTs performed within 3 months before or after ^68^Ga-PSMA PET/CT were reexamined for comparison. Outcome measurements are provided as median and range.

### Eligibility for ^177^Lu-PSMA-617

The multidisciplinary tumor board decided whether patients were eligible for treatment with ^177^Lu-PSMA-617. Patients were deemed eligible if metastases were predominantly PSMA positive on ^68^Ga-PSMA PET, PET scans were assessed visually, and no pre-defined SUV cut-off values were used to decide whether or not to treat patients.

## Results

### Patient characteristics

Five patients with RAI-refractory DTC underwent ^68^Ga-PSMA PET/CT to determine whether they were eligible for therapy with ^177^Lu-PSMA-617. Four patients had papillary thyroid carcinoma (PTC), and one patient had the follicular variant of papillary thyroid carcinoma (FvPTC). The median age at diagnosis was 50 years (range 39–65 years). Patient characteristics and previous treatments are summarized in Table 1. All patients developed locoregional lymph node metastases and distant metastases, over a median time span of 3.3 years (range 0–3.5 years) and 3.5 years (range 0–19.3) after the first diagnosis, respectively. Local recurrence was seen in two patients, after 3.6 and 10.2 years.

**Table 1:** Patient characteristics

No.	Sex	Age at diagnosis	Year of diagnosis	Type	TNM	Stage	Treatment history	
							Type	Number of treatments
1	♂	44	2008	FvPTC	T1aN0M+	II	Surgery	2
							I-131 ablation	1
							I-131 therapy	5
							Radiotherapy	2
2	♀	65	2008	PTC	T2N0M0	I	Surgery	6
							I-131 ablation	1
							I-131 therapy	1
							Radiotherapy	2
							^177^Lu-PSMA-617	2
							Lenvatinib	-
3	♀	39	2013	PTC	T3N0M0	I	Surgery	3
							I-131 ablation	1
							I-131 therapy	2
							Radiotherapy	1
4	♀	59	2010	PTC	T4aN1M0	III	Surgery	7
							I-131 ablation	1
							Radiotherapy	1
							Lenvatinib	-
5	♀	50	1996	PTC	T2N0M0	I	Surgery	8
							I-131 ablation	1
							I-131 therapy	2
							^177^Lu-PSMA-617	2
							Radiotherapy	1

### ^68^Ga-PSMA PET/CT

All five patients showed PSMA tracer uptake in known distant metastases. Two scans revealed metastases in formerly unknown sites. Local recurrence was found in one ^68^Ga-PSMA PET/CT.

The median SUV_max_ in cervical lymph nodes and distant metastatic disease was 3.59 (range 2.90–5.13) and 4.06 (range 0.85–10.56), respectively. SUV_max_ of local recurrence was 1.38. In Table 2, the median SUV_max_ of the various sites is shown.

**Table 2:** Results of ^68^Ga-PSMA PET/CT and ^18^FDG PET/CT

No.	Primary tumor	Moment of scan	Site	(Median) SUV_max_		Range
				PSMA	FDG	
1	2008	2017	No pathological uptake		–	–
		2018	**Leptomeningeal/drop metastases Th10**	**2.66**		2.66
		2018	No pathological uptake	–		–
2	2008	2017	Lungs		5.02	4.46–5.35
			Th4		2.94	2.94
		2017	Lungs		5.45	4.54–7.99
			Th4		2.79	2.79
		2017	**Left cervical lymph nodes**	**3.33**		3.04–3.61
			Lungs	8.03		7.65–9.40
			**Liver**	**7.18**		5.84–8.52
		2017	Left cervical lymph nodes	3.82		2.90–4.73
			Decrease in lungs	8.67		6.56–10.56
			Decrease in liver	6.01		6.01
		2018	Unchanged left cervical lymph nodes		4.20	4.00–2.40
			Increase in lungs		5.44	5.07–8.16
3	2013	2016	Lungs		1.85	0.93–2.66
		2017	Lungs	3.61		1.31–4.66
		2018	Recurrence/metastasis central compartment		3.61	3.61
			Lungs		3.21	2.27–4.89
4	2010	2016	Residual thyroid bed		4.55	4.55
			Lungs		4.44	2.51–7.51
			Mediastinal/hilar lymph nodes		15.31	11.03–26.24
			Lymph node m. pectoralis		1.93	1.93
			Lymph node parasternal		1.37	1.37
		2016	Residual thyroid bed	1.38		1.38
			Lungs	1.54		1.28–1.79
			Mediastinal/hilar lymph nodes	2.42		2.26–6.97
			Lymph node m. pectoralis	0.85		0.85
5	1996	2016	Right cervical lymph nodes	4.35		3.56–5.13
			Lungs	1.73		1.44–3.39
		2017	Right cervical lymph nodes		3.35	2.12–8.36
			Retropharyngeal		5.43	5.43
			Lungs		2.85	1.83–3.76
		2017	Right cervical lymph nodes		3.99	3.57–4.50
			Retropharyngeal		5.82	5.82
			Lungs		3.44	2.49–5.49

Entries in bold are newly diagnosed on ^68^Ga-PSMA PET/CT

### Treatment with ^177^Lu-PSMA-617

Based on the ^68^Ga-PSMA PET/CTs, three patients were deemed eligible for treatment with ^177^Lu-PSMA-617, of whom two patients underwent two cycles of 6000 MBq ^177^Lu-PSMA-617. Patient 2 and patient 5 had a time interval of 6 and 11 weeks between the two cycles, respectively.

### Case series

#### Patient 1: male, FvPTC since 2008, 44 years old at time of diagnosis

**Fig. 1:**
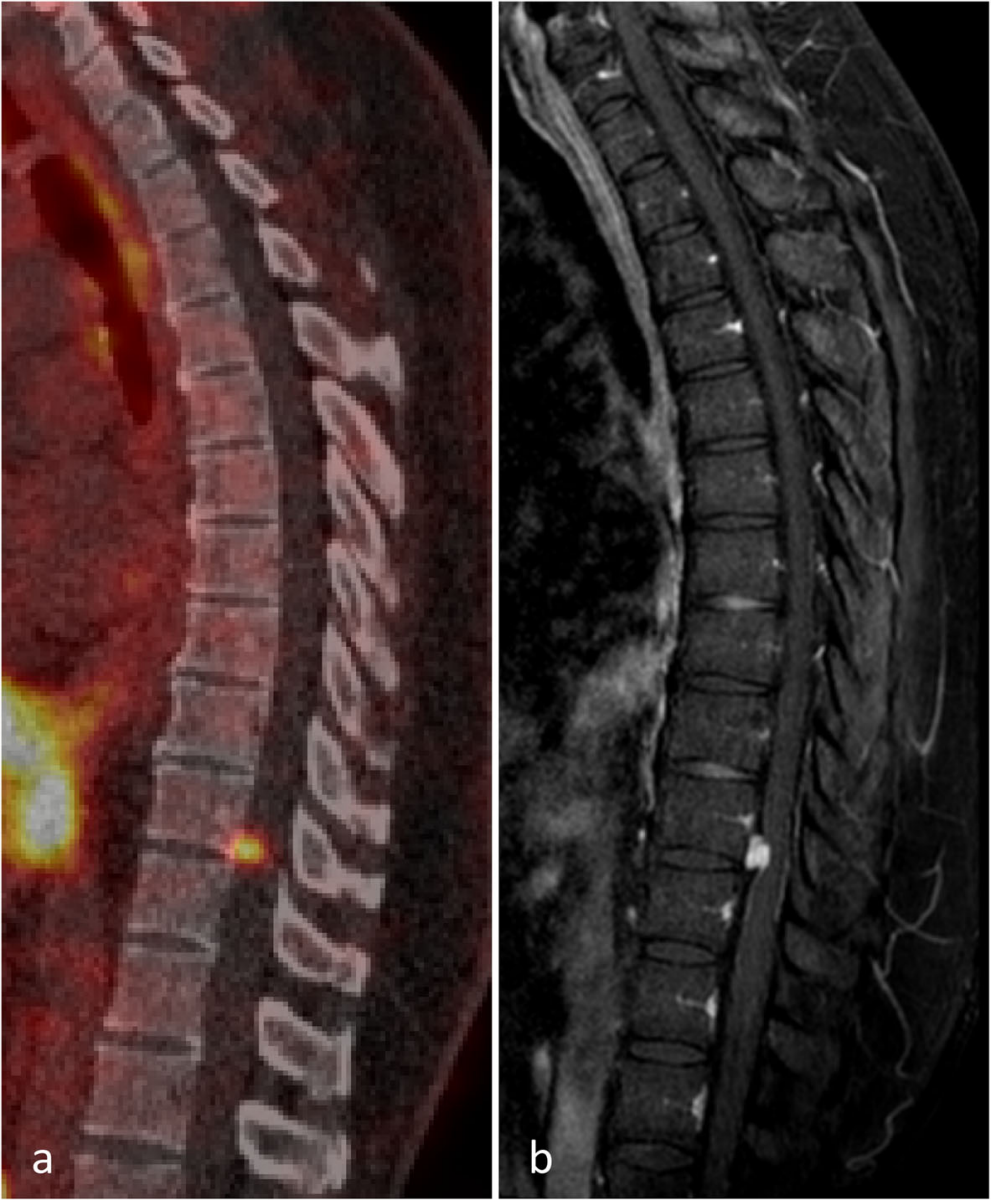
Images of patient 1. **a** Uptake dorsal of the tenth thoracic vertebra suspicious for leptomeningeal/drop metastasis on ^68^Ga-PSMA PET/CT. **b** Confirmation of metastasis on MRI

Six weeks prior to the ^68^Ga-PSMA PET/CT, a ^18^FDG PET/CT was made. On neither imaging tracer, uptake around a known metastasis in the fourth cervical vertebra was seen. However, as shown in Fig. 1a, intense uptake on the ^68^Ga-PSMA PET/CT was seen dorsal of the tenth thoracic vertebra, suspicious for leptomeningeal/drop metastasis. This lesion was not visible on the ^18^FDG PET/CT but was confirmed by MRI. The ^68^Ga-PSMA PET/CT showed high PSMA uptake making the patient eligible for ^177^Lu-PSMA-617 therapy. However, because of threatening spinal cord compression, this lesion was treated with stereotactic radiotherapy. Afterwards, on a second ^68^Ga-PSMA PET/CT, previous uptake disappeared. Therefore, the patient was no longer eligible for treatment with ^177^Lu-PSMA-617.

#### Patient 2: female, PTC since 2008, 65 years old at time of diagnosis

^68^Ga-PSMA PET/CT showed medium-high PSMA uptake in pulmonary metastases, which were also seen 5 weeks earlier on ^18^FDG PET/CT. Additionally, new hotspots were seen on ^68^Ga-PSMA PET/CT in the left side of the neck and new spots with high and medium uptake in the liver, both sites suspicious for metastases. Considering these findings, radioligand therapy with ^177^Lu-PSMA-617 was started. As shown in Table 2, the SUV_max_ of the pulmonary metastases on the ^68^Ga-PSMA PET/CT was higher than that on the ^18^FDG PET/CT. On the contrary, the ^18^FDG PET/CT showed tracer uptake in the earlier irradiated metastasis on the level of the fourth thoracic vertebra, whereas this lesion was not visible on the ^68^Ga-PSMA PET/CT.

**Fig. 2:**
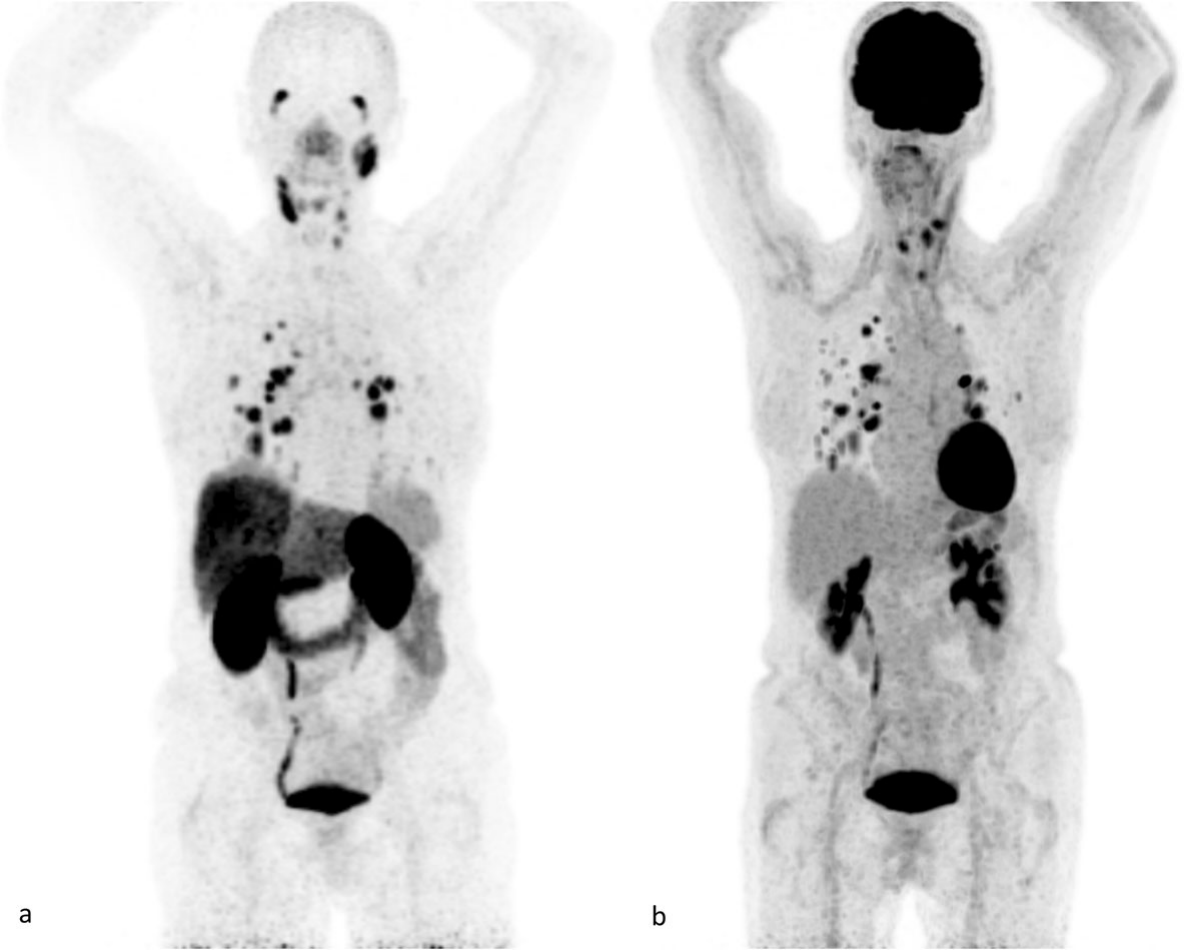
Images of patient 2. **a** Maximum intensity projection of ^68^Ga-PSMA PET/CT. **b** Uptake in multiple pulmonary metastases and cervical lymph nodes on ^18^FDG PET/CT

**Fig. 3:**
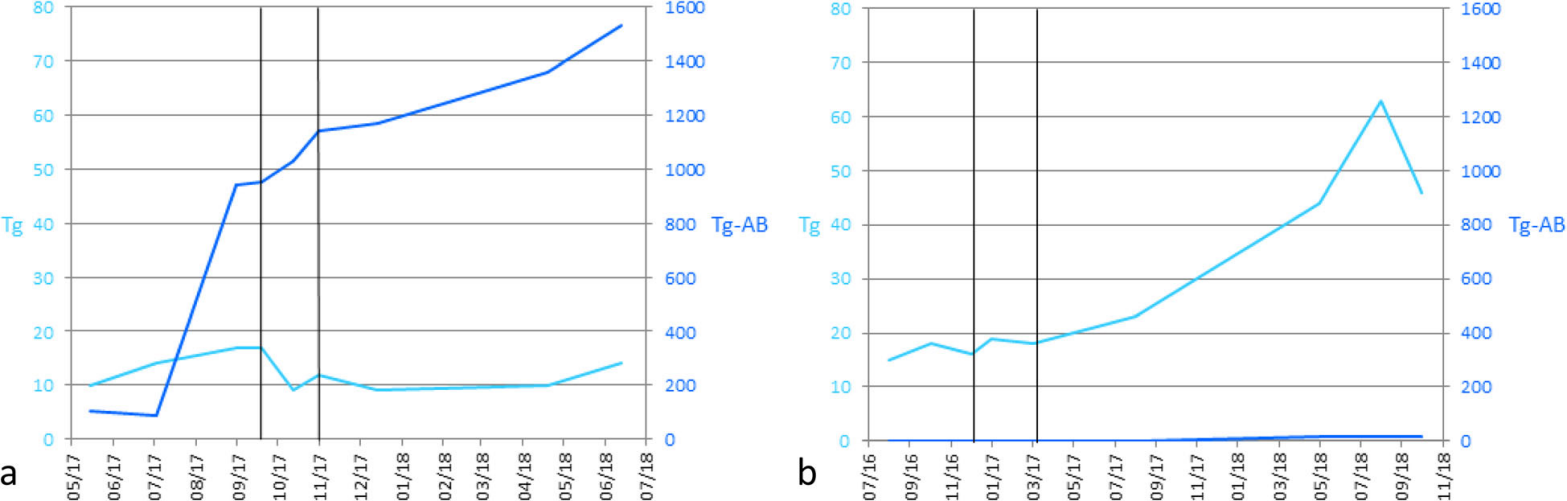
Tg and Tg antibody (Tg-AB) levels. Black vertical lines indicate the moment of treatment with ^177^Lu-PSMA-617. **a** Patient 2. **b** Patient 5

The post-therapy ^68^Ga-PSMA PET/CT was made 5 weeks after the second treatment with 6000 MBq ^177^Lu-PSMA-617 and demonstrated slight response. Still, multiple metastases in the lung were present of which few had decreased in size. Cervical metastases were stable under therapy. Liver metastases responded; one hotspot disappeared, the other decreased in size, and focal pathological PSMA uptake was reduced. As shown in Fig. 3a, a transient decrease in Tg values was seen while Tg antibodies increased. However, 7 months after treatment, progression was observed on imaging, as shown in Fig. 2. Also, Tg levels increased from 9 to 14 μg/L. Treatment with Lenvatinib was started later. Images of the immunohistochemical staining for PSMA in tumor tissue of patient 2 are shown in the Supplementary figure.

#### Patient 3: female, PTC since 2013, 39 years old at time of diagnosis

^68^Ga-PSMA PET/CT showed slow disease progression in the lungs. Only six of the dozens of metastases showed PSMA uptake, which was not enough to be deemed eligible for therapy with ^177^Lu-PSMA-617. This patient subsequently underwent radiotherapy of the right side of the neck.

#### Patient 4: female, PTC since 2010, 59 years old at time of diagnosis

PSMA tracer uptake varied in the known lymph node metastases, pulmonary metastases, and in local recurrence as shown in Table 2. Uptake was considered not enough to be eligible for ^177^Lu-PSMA-617 therapy. There were no signs of new metastases. No changes were observed compared to the ^18^FDG PET/CT 2 months earlier: in size, the lesions were unchanged; however, the SUV_max_ was lower in all lesions on the ^68^Ga-PSMA PET/CT relative to the ^18^FDG PET/CT; in the thyroid lesion and the parasternal lymph node metastasis, there was only slight tracer uptake. Later, disease progressed, including pleuritis carcinomatosa. Treatment with Lenvatinib was started.

#### Patient 5: female, PTC since 1996, 50 years old at time of diagnosis

^68^Ga-PSMA PET/CT showed uptake in lymph node metastases in the right side of the neck. The majority of pulmonary metastases showed PSMA uptake. ^177^Lu-PSMA-617 was started. A ^18^FDG PET/CT, 1 month after the second treatment with ^177^Lu-PSMA-617, showed disease progression. In the months following treatment, Tg values increased from 18 to 63 μg/L, as shown in Fig. 3b. A ^18^FDG PET/CT 6 months after treatment did not show further progression. Images of the immunohistochemical staining for PSMA in tumor tissue of patient 5 are shown in the Supplementary figure.

## Discussion

This study describes our first experience with ^68^Ga-PSMA PET/CT imaging and subsequent therapy of metastatic RAI-refractory DTC using ^177^Lu-PSMA-617. ^68^Ga-PSMA PET/CT detected various types of lesions including lymph node, pulmonary, and bone metastases. This is in concordance with Taywade et al*.*, Verma et al*.,* and Lütje et al*.*, who also detected these and various other lesions [14, 15, 19]. Furthermore, our results show uptake in liver metastases, which was also shown in RAI-sensitive patients by Verma et al*.* [15]. In addition, we found PSMA uptake in leptomeningeal metastases. Our results, although based on only five patients, indicate the potential clinical usefulness of ^68^Ga-PSMA PET/CT; not only because it is able to depict tumor lesions in various locations, but also because it may detect lesions which are not picked up by ^18^FDG PET/CT. Furthermore, ^68^Ga-PSMA PET/CT can be used for patient selection for therapy with ^177^Lu-PSMA-617.

Treatment with ^177^Lu-PSMA-617 has been proven to be safe and effective in patients with prostate carcinoma. Hofman et al*.* showed a PSA decline of 50% or more in 57% of patients and an objective response in 82% of patients with measurable disease, after administering up to 4 cycles with a mean radioactivity of 7.5 GBq with relatively mild side-effects [20]. Because of PSMA uptake in DTC metastases, treatment with ^177^Lu-PSMA-617 in RAI-refractory DTC was initiated. Patients with intermediate to intense PSMA uptake in all known lesions are most eligible for treatment with ^177^Lu-PSMA-617 [14]. In our study, two patients underwent this therapy. Patient 2 showed slight, temporary response on imaging, while Tg values decreased from 17 to 9 μg/L. However, Tg antibodies increased simultaneously. In patient 5, the lesions increased on imaging and Tg levels rose after ^177^Lu-PSMA-617 therapy. Although in patient 2 the ^177^Lu-PSMA-617 therapy shows some potential, data of more patients is needed to draw conclusions on the effectiveness of this therapy.

In prostate carcinoma, PSMA expression is found mostly in the cytoplasm and membrane of the epithelium. This is in contrast to most other solid tumors, where PSMA is expressed predominantly in the endothelium of the neovasculature [21]. Immunohistochemical expression of PSMA has previously been investigated in various forms of thyroid cancer. Lodewijk et al*.* found PSMA positivity in neovasculature in more than 92% of medullary thyroid carcinoma, while the tumor cells were PSMA negative [22]. Heitkötter et al*.* studied PSMA on IHC in PTC and follicular thyroid carcinoma and found positivity in tumor-associated neovasculature in 18 out of 31 and 4 out of 10 patients, respectively [23]. Moore et al*.* investigated 37 patients and found PSMA significantly overexpressed in the neovasculature of DTC, particularly in RAI-refractory patients and in distant metastases. No PSMA expression was seen on the tumor cells [13]. Recently, in a study including 59 DTC patients, Sollini et al. found PSMA positivity in microvessels in 80%. Of these cases, 53% was moderately positive and 47% highly positive [24]. In conclusion, the localization of PSMA expression in the neovasculature of thyroid carcinoma instead of in tumor cells as in prostate carcinoma might explain why ^177^Lu-PSMA treatment is less effective in DTC than in prostate carcinoma.

Interestingly, PSMA uptake was heterogeneous in our patients. Since PSMA expression in thyroid carcinoma is a relatively new finding, biological behavior needs to be further investigated. We hypothesize that the amount of proliferation may vary in different lesions, which influences the amount of vascularization. As PSMA expression is seen in neovasculature, this might explain the heterogeneous uptake.

## Conclusion

For patients with RAI-refractory DTC, ^68^Ga-PSMA PET/CT can be valuable for staging, as it is able to detect various types of lesions and may detect lesions which are not picked up by ^18^FDG PET/CT. Furthermore, ^68^Ga-PSMA PET/CT might be used to identify patients for treatment with ^177^Lu-PSMA-617. One of the two patients who underwent ^177^Lu-PSMA-617 therapy showed slight, temporary response. To draw conclusions on the effectiveness of this therapy, evaluation of more patients is needed.

## Supplementary information

**Additional file 1.** Supplementary figure Images of immunohistochemical staining for PSMA in tumor tissue of patients who were eligible for ^177^Lu-PSMA-617 therapy, at 10 times and 400 times magnification. In both patients tumor cells did not express PSMA, while the neovasculature was PSMA positive. a Patient 2: extensive PSMA positivity in the stroma (probably endothelium) in between the tumor cells. b Patient 5: PSMA positivity in the endothelium of the fibrovascular cores of the papillary proliferations.

## Data Availability

The datasets used and/or analyzed during the current study are available from the corresponding author on reasonable request.
