# Supplementary figures and images for: 68Ga-PSMA PET/CT in radioactive iodine-refractory differentiated thyroid cancer and first treatment results with 177Lu-PSMA-617
Source: EJNMMI Res. 2020 Mar 6;10:18. doi: 10.1186/s13550-020-0610-x (PMC7060303; doi:10.1186/s13550-020-0610-x)

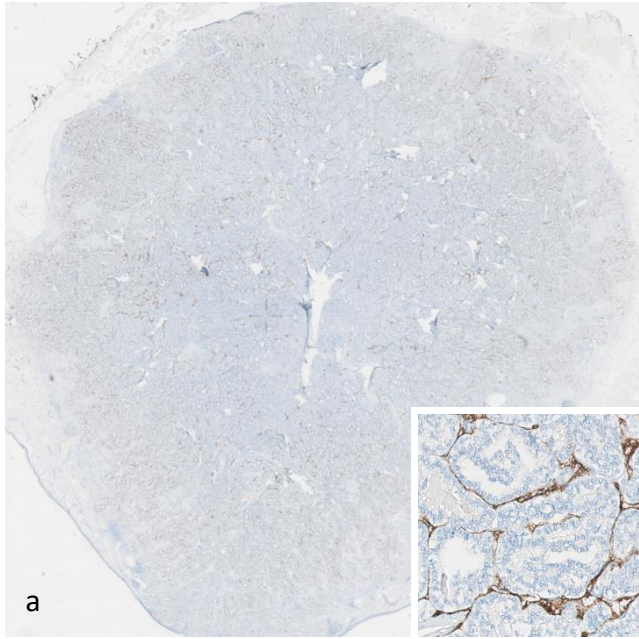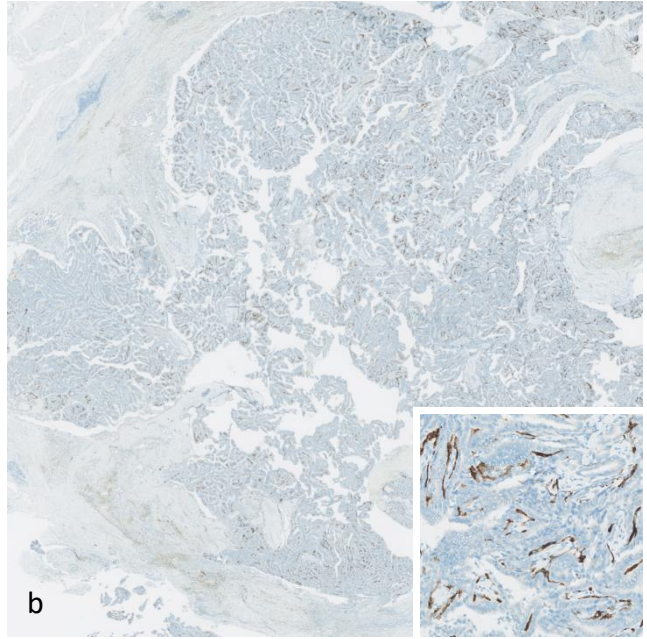

Supplement: Supplementary file 1 — Additional file 1. Supplementary figure Images of immunohistochemical staining for PSMA in tumor tissue of patients who were eligible for 177Lu-PSMA-617 therapy, at 10 times and 400 times magnification. In both patients tumor cells did not express PSMA, while the neovasculature was PSMA positive. a Patient 2: extensive PSMA positivity in the stroma (probably endothelium) in between the tumor cells. b Patient 5: PSMA positivity in the endothelium of the fibrovascular cores of the papillary proliferations. [file 13550_2020_610_MOESM1_ESM.pdf]
